# Common Bile Duct Stones ERCP or Surgery?

**DOI:** 10.1155/1992/14576

**Published:** 1992

**Authors:** P. C. Bornman

**Affiliations:** Department of Surgery University of Cape Town Medical School Observatory Cape Town 7925 South Africa


alter the natural history of the disease, and delay or prevent the development or cirrhosis. Data from our unit as well as others suggests that to be the case. The current series, many of whom have end stage disease, cannot answer that question. This series is a large group of ill patients with end stage disease, and the authors are to be complimented on their results with this extremely difficult high risk group.

John L. Cameron
The Johns Hopkins University
School of Medicine
Richard Starr Ross Research Building
720 Rutland Avenue
Room 759
Baltimore
Maryland 21205-2196
United States of America

### REFERENCES

1. Helzberg, J.H., Petersen, J.M. and Boyer, J.L. (1987) Improved survival with primary sclerosing cholangitis. *Gastroenterology*, **92**, 1869–1875
2. Cameron, J.L., Pitt, H.A., Zinner, M.J., Herlong, H.F., Kaufman, S.L., Boitnott, J.K. and Coleman, J. (1988) Resection of hepatic duct bifurcation and transhepatic stenting for sclerosing cholangitis. *Annals of Surgery*, **207**, 614–622
3. Hutson, D., Russell, E., Jeffers, L., *et al.* (1986) Balloon dilation of biliary strictures in primary sclerosing cholangitis through a cholodochojejuno-cutaneous fistula: a new therapeutic approach (abstract). *Gastroenterology*, **90**, 1470
4. Marsh, J.W., Iwatsuki, S., Makowka, L., *et al.* (1988) Orthotopic liver transplantation for primary sclerosing cholangitis. *Annals of Surgery*, **207**, 21–25

### COMMON BILE DUCT STONES : ERCP OR SURGERY?

### ABSTRACT

*Stain, S.C., Cohen, H., Tsuishoysha, M., Donovan, A.J. (1991) Choledocholithiasis . Endoscopic sphincterotomy or common bile duct exploration. Annals of Surgery; 213: 627–634*

**A prospective randomized trial was conducted of preoperative endoscopic sphincterotomy and surgery (ES&S) or surgery alone (SA) in 52 patients with cholecystolithiasis and choledocholithiasis that were candidates for elective surgery. After ES&S**

**65% of patients were stone free. Eighty-eight per cent of patients with SA were stone free after surgery ($p < 0.05$). Three patients in each group had residual stones at the completion of the operation. Five of these six had more than 20 common bile duct (CBD) stones. There was one episode of major hemorrhage in a patient in each group and no deaths. Costs were essentially equal for the individual patient with a successful ES as compared to SA. Societal costs of a program of preoperative endoscopic retrograde cholangiopancreatography and ES would be higher because of the cost of screening for patients with CBD stones. These results do not support preoperative ES as a technique for clearance of the CBD of stones on the basis of efficacy, morbidity rate, or cost.**

### PAPER DISCUSSION

KEY WORDS: Endoscopic sphincterotomy, common bile duct, gallstones

This is the second prospective randomised study which has failed to demonstrate any clear benefit of endoscopic removal of common bile duct (CBD) stones prior to cholecystectomy (ES & S) over the conventional surgical approach. As in the larger Leicester study[1] morbidity and mortality was similar in patients who underwent the seemingly less invasive endoscopic method of removal of CBD stones. The high failure rate of endoscopic removal of CBD stones (35%), and the calculated higher cost of preoperative screening for CBD stones were the main reasons why the authors do not support routine endoscopic removal of CBD stones before cholecystectomy in surgically fit patients.

The apparent failure of endoscopic removal of CBD stones to confer benefit to patients undergoing cholecystectomy is surprising and somewhat disappointing, as this approach has several potential benefits for the patient. Firstly, the knowledge of the number and situation of stones and possible associated bile duct anomalies (or other pathology) may provide useful information to the surgeon in planning his operative strategy. Secondly, successful removal of stones prior to cholecystectomy should make the operation simpler and safer by avoiding common bile duct exploration. Thirdly, in patients who present with jaundice and cholangitis, cholecystectomy may be deferred until their general condition has been optimised. Finally, in elderly patients, surgery may be delayed indefinitely, as the risk of developing life threatening complications related to the intact gallbladder is minimal after successful endoscopic clearance of the bile duct.

Perhaps implicit in these potential benefits of preoperative ERCP, ES and removal of CBD stones, is the explanation why in both studies patients undergoing the endoscopic method of removal of CBD stones did not fair better than those who were treated with the conventional surgical approach. In patients who were randomised to surgery alone, surgeons had the benefit of knowing exactly what the status of the CBD pathology was prior to surgery. On the other hand, patients in the ES & S arm of the study may not have derived the full advantage of this approach. Most patients underwent surgery early and during the same admission. It is conceivable that a number of patients who had a successful clearance of their bile ducts could have benefited from delaying surgery; e.g. those who presented with jaundice with or without cholangitis or the elderly patient with reversable associated medical conditions. Unfortunately these details are not given in either of the two randomised studies.

The high failure rate of endoscopic clearance of CBD stones in this study requires further comment. In addition to the strict criteria used for CBD clearance by the authors, the number of patients with innumerable stones (defined as more than 20 stones) appeared to be considerably higher than in other reported series. We are not given the exact number and whether they were equally distributed between the two treatment groups, but this may offer an explanation for the lower success rate in this study. It is also possible that a second endoscopic attempt at removal of the stones could have improved the success rate, albeit at an increased cost.

The comparison between the success rate of endoscopie and surgical removal of CBD stones was somewhat artificial since all patients wre scheduled to undergo surgery. The results can therefore be interpreted in a more positive way by concluding that preoperative andoscopic removal of CBD stones significantly reduced the need for CBD exploration; 12/26 (ES & S) vs 23/26 surgery alone ($p < 0.003$). It should also be emphasised that the incidence of retained stones after surgery was similar in the two treatment groups and that subsequent endoscopic removal of stones avoided further surgery in all patients.

The total incidence of retained stones after surgery in both arms of this study was high (6/35 = 17%) and occurred mostly in patients with innumerable stones. These could have been avoided by a biliary drainage procedure but as the authors point out, surgeons now rely increasingly on ES in this situation. Long-term follow-up in this subgroup of patients is needed to determine the wisdom of this approach.

It is interesting to note that few septic complications occurred in this study despite the relatively high failure rate of ES. This is contrary to the experience reported in the Leicester study[1] where most of the severe complications occurred in the small number of patients in whom endoscopic clearance of CBD stones failed. There is no apparent reason for the discrepancy between these two studies except that patients in the Leicester study were much older, increasing their risk for sepsis.

Although the numbers were rather small in both studies, it is unlikely that a significant difference would emerge with larger numbers, given the low morbidity and mortality in both treatment groups. There are several other recent series which have also reported a low morbidity and mortality for surgical removal of CBD stones[2-4]. Although it appears that the endoscopic route reduces operating time and perhaps hospital stay, it remains difficult to calculate and compare cost benefits in different countries[5].

It must be emphasised that both randomised studies only compared the outcome of endoscopic and surgical management in patients with proven CBD stones. This does not include the large number of patients with gallbladder stones and suspected (but unproven) CBD stones, a problem which is frequently encountered in practice. The detection of CBD stones with ultrasound and CT remains far from satisfactory in patients who present with clinical and biochemical evidence of biliary obstruction. Some patients may harbour other pathology which can mimic stones. In this respect ERCP is superior to any other test in delineating the pathology in biliary obstruction. Therefore, randomisation in this setting before ERCP is carried out, should give a better evaluation of its diagnostic and therapeutic value in patients who are to undergo biliary surgery.

Professor P.C. Bornman
Department of Surgery
University of Cape Town Medical School
Observatory 7925
Cape Town
South Africa

### REFERENCES

1. Neoptolemos, J.P., Carr-Locke, D.L. and Fossard, D.P (1987) Prospective randomised study of pre-operastive endoscopic sphincterotomy versus surgery alone for common bile duct stones. *British Medical Journal*, **294**, 470–474
2. Neoptolemos, J.P., Davidson, B.R., Shaw, D.E. *et al.*, (1987) Study of common bile duct exploration and endoscopic sphincterotomy in a consecutive series of 438 patients. *British Journal of Surgery*, **74**, 916–921
3. Muller, B.M., Kozarek, C.A., Ryan, J.A. Jr. *et al.*, (1988) Surgical versus endoscopic management of common bile duct stones. *Annals of Surgery*, **207**, 135–141
4. Pappas, T.N., Slimane, T.B. and Brookes, D.C. (1990) 100 Consecutive common duct explorations without mortality. *Annals of Surgery*, **211**, 260–262
5. Van Stiegmann, G., Pearlman, N.W., Goff, J.S., Sun, J.H. and Nortone, C.W. (1989) Endoscopic cholangiography and stone removal prior to cholecystectomy. *Archives of Surgery*, **124**, 787–790

### DOES METOCLOPRAMIDE REDUCE VARICEAL PRESSURE?

### ABSTRACT

*Kleber, G., Sauerbruch, T., Fischer, G., Geigenberger, G., Paumgartner, G. (1991) Reduction of transmural oesophageal variceal pressure by metoclopramide. Journal of Hepatology;* **12**: 362–366.

**In nineteen patients with portal hypertension and oesophageal varices, transmural variceal blood pressure was determined endoscopically by direct puncture of the varices before and after intravenous administration of 20 mg metoclopramide or placebo. No change in pressure was observed after placebo (mean difference −1.3 ± 24.5%, N.S.), however, metoclopramide reduced the pressure by 17.6 ± 18.6% ($p$ = 0.02). Our results suggest that metoclopramide may be beneficial for the prevention of treatment of variceal haemorrhage.**

